# Role of transcription factor-mediated nucleosome disassembly in *PHO5* gene expression

**DOI:** 10.1038/srep20319

**Published:** 2016-02-04

**Authors:** Hungyo Kharerin, Paike J. Bhat, John F. Marko, Ranjith Padinhateeri

**Affiliations:** 1 Department of Biosciences and Bioengineering, Indian Institute of Technology Bombay, Mumbai 400076, India; 2 Department of Physics, Department of Molecular Biosciences, Northwestern University, Evanston, IL.

## Abstract

Studying nucleosome dynamics in promoter regions is crucial for understanding gene regulation. Nucleosomes regulate gene expression by sterically occluding transcription factors (TFs) and other non–histone proteins accessing genomic DNA. How the binding competition between nucleosomes and TFs leads to transcriptionally compatible promoter states is an open question. Here, we present a computational study of the nucleosome dynamics and organization in the promoter region of *PHO5* gene in *Saccharomyces cerevisiae*. Introducing a model for nucleosome kinetics that takes into account ATP-dependent remodeling activity, DNA sequence effects, and kinetics of TFs (Pho4p), we compute the probability of obtaining different “promoter states” having different nucleosome configurations. Comparing our results with experimental data, we argue that the presence of local remodeling activity (LRA) as opposed to basal remodeling activity (BRA) is crucial in determining transcriptionally active promoter states. By modulating the LRA and Pho4p binding rate, we obtain different mRNA distributions—Poisson, bimodal, and long-tail. Through this work we explain many features of the *PHO5* promoter such as sequence-dependent TF accessibility and the role of correlated dynamics between nucleosomes and TFs in opening/coverage of the TATA box. We also obtain possible ranges for TF binding rates and the magnitude of LRA.

The eukaryotic chromatin in gene-rich regions resembles “beads on a string” made of nucleosomes—a DNA-protein complex with 147 base pairs (bp) of DNA wrapped around an octameric histone protein in nearly two turns^1^,^2^. Apart from nucleosomes, chromatin also consists of large number of non–histone proteins such as transcription factors (TFs). All these proteins, including nucleosomes, can constantly dissociate and bind back onto the DNA giving rise to a highly dynamic structure of chromatin. Research has shown that nucleosomes are not positioned at random locations on the genome but have a specific organization that may have functional relevance^3^,^4^,^5^,^6^. It is becoming increasingly clear that one major role of nucleosomes is to regulate access to certain parts of the DNA (e.g., TATA box) by sterically occluding them^7^,^8^,^9^. This is crucial for many cellular processes like gene transcription, DNA replication^10^, and repair that require physical access to bare DNA.

One important effort in chromatin research in the past one or two decades has been to understand the precise organization of nucleosomes along the DNA and to examine what controls their positioning on the DNA^3^,^4^,^5^,^6^. It has been established that some of the important factors that contribute towards nucleosome positioning and organization are ATP-dependent chromatin remodeling machines, DNA sequence, chemical modification of histones and DNA (acetylation/methylation), and the presence of certain non–histone proteins. ATP-dependent remodeling machines are known to reposition nucleosomes by disassembling and sliding them along the DNA^11^,^12^. In the absence of the appropriate function of such machines (or equivalently in the absence of ATP) it has been seen that nucleosomes are not well positioned at many of the crucial locations^6^,^13^,^14^. Starting with that of Kornberg and Stryer, a number of studies have shown that a “barrier” (which is either a strongly bound nucleosome/non–histone protein-complex or a nucleosome-disfavoring DNA sequence^5^) can cause periodic organization of nucleosomes, which is known as statistical positioning^3^,^15^,^16^. Many studies, in particular recent studies by Widom, Segal and others, have suggested that nucleosome positioning depends on the DNA sequence where nucleosome-favoring sequences often have a specific oscillatory placement of AA/TT/TA and GC dinucleotides^4^,^17^,^18^. It has been found that another way of controlling nucleosome positioning and dynamics is to make chemical modifications either on the DNA (e.g., DNA methylation^5^,^19^) or on histone proteins (e.g., acetylation of histone tails^5^,^20^).

Given that a large number of factors can influence nucleosome positioning, its organization is highly diverse and varies considerably from gene to gene and location to location^8^,^9^. As a result of this, predicting nucleosome organization accurately at specific locations is still an extremely difficult task. One of the crucial locations where nucleosome organization is least understood is the promoter region of genes. Given that nucleosome positioning at promoters can essentially act as a switch to turn on or off the gene, experimenters have been trying to understand nucleosome positioning at the promoter regions. In constitutive promoters—promoters of genes that are always in the ‘ON’ state—one typically finds nucleosome free regions (NFR) of size ≈150–200 bp adjacent to transcription start sites (TSS)^21^,^22^. However, for inducible promoters—promoters of genes that can be switched on and off based on various signals—one typically does not find a bona fide NFR as seen in constitutive promoters^21^,^22^. The nucleosome positioning for inducible promoters can be more complex and is not well understood.

In yeast, a number of experiments have been carried out to investigate nucleosome positioning at various inducible promoters. For example, in *GAL1*/ *10* promoter, interesting interplay between nucleosomes, remodelers, and TFs are known to lead to strong nucleosome positioning^8^. In this process, a RSC–nucleosome complex “barrier” is formed and is thought to facilitate transcriptional activator (Gal4p) binding. Post induction, the Gal4p is thought to recruit SWI/SNF remodelers and remove the nucleosomes adjacent to the barrier^23^. In the cell-cycle-regulated *HO* and *CLN2* promoters, the binding of activator SBF at their binding sites has been shown to correlate with nucleosome eviction and gene expression^24^,^25^. Another classic example of nucleosome-mediated gene regulation is that of *PHO5*, a gene that encodes acid phosphatase required for scavenging phosphate from the environment. Typically, nucleosomes at the *PHO5* promoter are known to form a regular array with relatively high occupancy^26^,^27^. However, this array is disrupted in the presence of activator Pho4p^28^ and gene expression ensues^29^,^30^,^31^. Thus, though different promoters have different architectures with respect to nucleosome occupancy and positioning along the DNA, one of the common features shared by these promoters is the activator-dependent nucleosome dynamics. The question is then how does the competition between activators and nucleosomes to occupy the same space on the promoter determines whether the gene is ON or OFF.

Even though many experimental studies^25^,^30^,^31^,^32^ have investigated the nucleosome dynamics in the promoters, there are no computational studies, to the best of our knowledge, that address how different competing factors work together in determining the nucleosome dynamics and organization in the promoters. TF binding, local DNA sequence, and ATP-dependent remodeling are all important factors that determine the nucleosome dynamics and influence the gene regulation. In the current work, using *PHO5* promoter of yeast *Saccharomyces cerevisiae* as a model system^33^, we investigate the nature of promoter dynamics (activation–inactivation) as a result of the interplay between all these different factors. We also investigate the effect of Pho4p and nucleosome dynamics on gene expression and transcriptional noise.

## Models and Methods

To study the complex problem of nucleosome dynamics in the presence of non–histone proteins (e.g., TFs), we have made a stochastic model that has three layers: (i) nucleosome kinetics with sequence- and ATP-dependent effects (ii) transcription factor kinetics and (iii) coupling between transcription factor and nucleosome kinetics via enhanced local remodeling of nucleosomes. In the paper, DNA is modeled as a one-dimensional (1D) lattice of N bp; nucleosomes and TFs are modeled as sterically interacting (hard-core) particles of size *k* bp and *k*_*t*_ bp, respectively.

### Nucleosome kinetics

**Figure 1:**
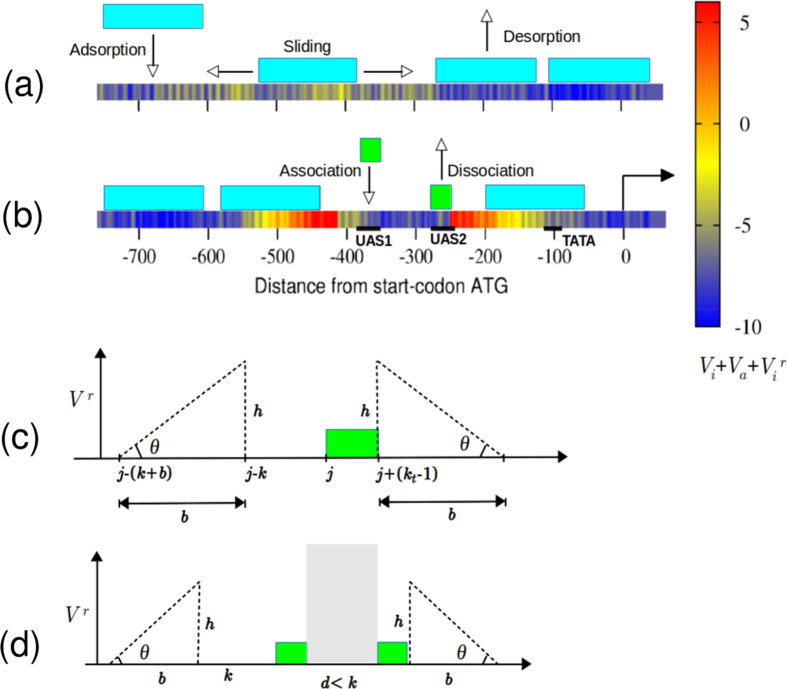
Model for nucleosome dynamics and local remodeling activity. (**a**) Nucleosome (cyan boxes) dynamics have three kinetics events: nucleosome binding, dissociation, and sliding. (**b**) In the *PHO5* promoter region, transcription activators (green boxes) can associate or dissociate with certain kinetic rates at specific-sites (UAS1 and UAS2). Regulatory elements, such as UAS1, UAS2, and the TATA are indicated. The arrow at the origin (0) indicates the start of the gene. The DNA is color-coded according to the nucleosome affinity given by the total potential energy. (**c**) When one transcription activator is bound at the *j*^*th*^ site, it modifies the potential energy by adding 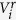 (remodeling potential giving rise to localised nucleosome removal) as shown here. (**d**) When two transcription activators are simultaneously bound at UAS1 and UAS2 (where the separation *d* < *k*), the potential 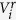 has the form shown here. The width of 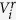 is taken as *b* = 150 bp and the slope (tan*θ*) is varied such that the height is given by *h* = *b*tan*θ*.

We assume that nucleosomes can have three kinetic moves—binding, dissociation and sliding—as discussed earlier in the literature^34^ (Fig. 1a). On the DNA, the histone octamer of size *k* bp can bind with an intrinsic rate *r*_on_ = *r*_0_. Since it is known that nearly any part of the genomic DNA can easily wrap around histones to form a nucleosome, we assume that the binding is sequence-independent. However, once bound, a nucleosome dissociates (disassembles) in a sequence-dependent manner. This dissociation occurs at a rate given by


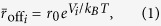


where *V*_*i*_ is the sequence-dependent binding energy at *i*^*th*^ position and *r*_0_ is the intrinsic removal rate per nucleosome. The potential energy function can be obtained using *V*_*i*_ = −*k*_*B*_*T*ln*P*_*i*_, where *P*_*i*_ is the equilibrium probability of finding a nucleosome at *i*^*th*^ bp in the limit of very low nucleosome density^4^,^5^. We obtain the values of *P*_*i*_’s by submitting a genomic sequence of our interest to the online server of Segal *et al.* (http://genie.weizmann.ac.il/software/nucleo_prediction.html) that computes the probabilities based on bioinformatic analysis of *in vitro* data. We set the nucleosome concentration low so that we obtain the potential “seen” by a single nucleosome. Given the binding energy function, the thermally driven adsorption–desorption process obeys the Boltzmann condition given by


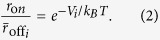


Based on experimental data, 〈*V*_*i*_〉 is estimated to be ≈−42 *k*_*B*_*T*, which is nothing but the average binding energy of histone octamer onto DNA^34^,^35^. Large negative *V*_*i*_ values implies that the the typical nucleosome removal rate 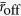 is very small, and thermal forces alone cannot displace nucleosomes within biologically relevant timescales. In the cells, the job of destabilising nucleosomes is performed by ATP-dependent remodeling machines^11^. The effect of ATPase activity is introduced into the model in two ways. First, ATP-dependent remodeling machines increase the dissociation rate such that^34^


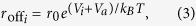


where *V*_*a*_ is the energy contribution from ATP-dependent “active” chromatin remodeling. Second, ATP-dependent machines can also slide the nucleosomes along the DNA. We assume each nucleosome is slid in a randomly chosen direction with a rate *α*_*s*_ and stops when it sterically interacts with its nearest neighbour (histone or transcription factor). We also assume that ATPases can slide nucleosomes independent of the local DNA sequence^36^. Since thermal sliding events are extremely rare, we neglect the possibility of any ATP-independent sliding events. We call the combined effects of ATP-assisted dynamics, *V*_*a*_ and *α*_*s*_, as basal remodeling activity (BRA). The underlying assumption here is that there is BRA all the time in the regions near TSS/promoters. This is the simplest kinetic model that would explain many of the experimentally seen phenomena. Our earlier theoretical work^34^,^37^, and some experimental work^6^ have shown that without ATP-dependent basal remodeling, one will not be able to obtain the well known “statistical positioning” of nucleosomes near TSS in the coding region.

### Pho4p interaction

Apart from nucleosomes, we have also introduced the binding–dissociation dynamics of the transcriptional activator, Pho4p, into the model. We consider Pho4p as a particle of size *k*_*t*_ = 17 bp, and assume that it can only bind at specific binding sites in the promoter region, marked as UAS1 and UAS2 in Fig. 1b. We also assume that Pho4p proteins bind independent of each other at a rate *r*_onp_ per binding site, whenever sterically permissible. The Pho4p at UAS1 and UAS2 dissociate with rates 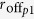 and 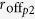, respectively. The fact that UAS2 has a higher affinity for the Pho4p is incorporated into the model by enforcing 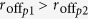
27,38. During the simulation, the Pho4p is introduced only after the nucleosome dynamics has reached a steady state (~1 hour of nucleosome-DNA simulation time^34^).

### Location-dependent active remodeling

So far we have only considered a basal ATP-dependent remodeling of nucleosomes—that is, the contribution to the active nucleosome removal *V*_*a*_ was independent of the location. However, experiments suggest that nucleosomes at the promoters are evicted/remodeled more often when compared to other regions^39^,^40^. Often such eviction/remodeling is a result of a series of signals^39^, for which binding of transcriptional activators is thought to be the primary step^29^. For example, transcriptional activators, once bound, are known to help recruit active remodelers that can dislodge nearby nucleosomes^29^,^41^. This is like a local remodeling activity (LRA) limited to the vicinity of the bound activators^42^. In our model, we account for this local activity by introducing a location-dependent potential 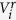 that is coupled to the TF binding. Once a transcription activator binds, the new nucleosome removal rate becomes


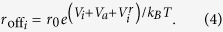


We introduce 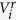 such that it is non-zero only in the vicinity of the bound Pho4p, as shown in Fig. 1c,d. In the absence of Pho4p, 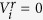. For simplicity, and motivated by forms of the potentials that create NFR^16^,^37^, we consider the following profile for 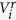 that decreases linearly with distance up to “*b*” bp:


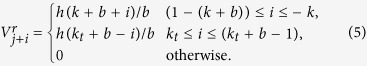


Here *h* is the maximal local remodeling energy and *b* is the width of the potential energy. This potential is “activated” only when at least one transcription factor is bound at location *j* (Fig. 1c), where *j* is the location of either at UAS1 or UAS2 (Fig. 1b,c). The local remodeling potential to the left side of the position *j* spans the region (*j* − *k* − 150) to (*j* − *k*) because this leads to removal of nucleosomes immediately to the left of the location *j*. When both UAS1 and UAS2 are occupied by activators, the resulting potential for local activity has the form shown in Fig. 1d. As in the previous case, this also leads to eviction of nucleosomes from the left and right of the activator binding sites. Note that the separation between UAS1 and UAS2 is smaller than the size of a nucleosome. Therefore, when Pho4p’s are bound, nucleosome is excluded from the region between UAS1 and UAS2. Conversely, when nucleosome occupies UAS1 and UAS2 at the same time, Pho4p cannot bind.

### Model for transcription

**Figure 2:**
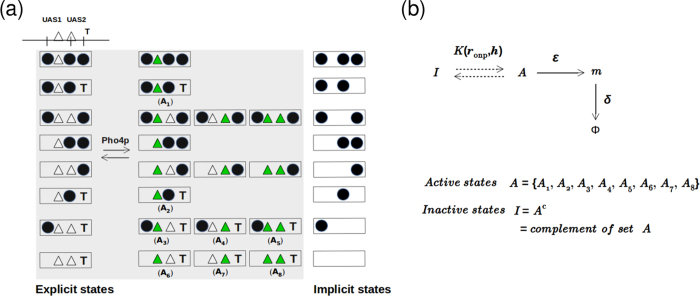
Transcription Model. (**a**) The competition between nucleosomes (filled circles) and Pho4p (filled triangles) proteins gives rise to different promoter states. At the top, we show a typical *PHO5* promoter region with “T” representing the TATA box, the two empty triangles representing the two Pho4p binding sites (UAS1 and UAS2) and the three vertical line segments representing reference locations for −1 (right), −2 (middle) and −3 (left) nucleosomes. When there are no Pho4p bound (both the triangles are empty), we can define eight different arrangements of nucleosomes (leftmost column) as done by Brown *et al.*^44^. These eight states are also depicted in the rightmost column without explicitly showing the binding sites of Pho4p (labelled as “implicit states”). The dark green (filled) triangles represent bound Pho4p proteins. When the presence/absence of 3 nucleosomes and 2 Pho4p proteins are explicitly accounted for, we can define 24 different states (see the shaded region labelled as “explicit states”). Among these 24 states, there are eight states where at least one Pho4p is bound and the TATA site is exposed. We define these states as “active” (*A*_*i*_) states. The rest are “inactive” states (*I*). (**b**) The dynamics of nucleosomes and Pho4p make the promoter switch between active and inactive states with some effective equilibrium constant which will depend on the protein binding rate (*r*_onp_) and local chromatin remodeling parameter (*h*). Promoter at the active state can produce mRNA (m) at the rate *ε* and the mRNA can decay at the rate *δ*.

We simulate a long region (5000 bp) around the promoter of the *PHO5* accounting for both nucleosome and TF kinetics as described above, using kinetic Monte Carlo simulations with Gillespie algorithm^43^. However, for the purpose of analysis, we will focus on a 600 bp promoter region adjacent to the TSS. In this region, as nucleosomes and activators bind and dissociate, competing for space on the DNA, there are many arrangements of nucleosomes and activators possible—each nucleosome moving by one base pair gives us a different microstate. For comparison with experiments, we bin these microstates to eight coarse-grained states as proposed by Brown *et al.*^44^ in their experiments (Fig. 2a (right)); these are promoter nucleosome states representing all possible configurations where −1, −2 and −3 nucleosomes are present/absent (see Supplementary Information for details). We call these “implicit” states since activator binding is not explicitly accounted for. However, since our aim in this paper is to study nucleosome dynamics in the presence of activators, we go beyond these nucleosome-only states and also incorporate different states that are possible from activator binding. We define twenty-four “explicit” states shown in Fig. 2a (left). Among these 24 states (configurations/arrangements), we can identify two kinds of macro-states—“active” (*A*) state and “inactive” (*I*) states. We consider a promoter state as active when at least one Pho4p is bound and the TATA binding site is empty (see eight states in Fig. 2a marked as *A*_1_, *A*_2_, *A*_3_, ..., *A*_8_). Any other promoter state is considered as inactive. Whenever the promoter is active, in the simulation, we produce mRNA at a constant rate *ε* (Fig. 2b). When the promoter is inactive, it cannot produce any mRNA. The produced mRNA can decay at a rate *δ* per mRNA. Let *m* be the number of mRNA molecules present at *t* = 1 hour after the addition of Pho4p proteins. From our simulations we computed the distribution of mRNA molecules *P*(*m*). We also computed the mean mRNA number 〈*m*〉, the variance *σ*^2^ = 〈*m*^2^〉 − 〈*m*〉^2^, and the Fano factor = *σ*^2^/〈*m*〉.

### Numerical parameters

The three kinetic parameters, namely, *r*_on_, 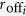 and *α*_*s*_ for nucleosome dynamics are determined from previous experiments, as done in the literature^34^,^35^. The rate of nucleosome adsorption is set to *r*_on_ = 12*s*^−1^. This value has been estimated from experiments using *Xenopus* egg extracts^35^ and has been used elsewhere^34^,^37^. As the desorption rate 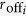 depends on ATPase parameter *V*_*a*_, we set *V*_eff_, the net effective binding potential experienced by a nucleosome, as *V*_eff_ = *V*_*a*_ + 〈*V*_*i*_〉; and use *V*_eff_ as a free parameter, where, 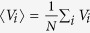 is the average potential energy over the naked DNA sequence of size *N* = 5000 bp of *PH05* gene when its full promoter region is taken into account. The active sliding parameter of the nucleosome is set to *α*_*s*_ = 0.0024*s*^−1^ based on the estimates of earlier works^34^,^45^. We fixed the size of nucleosome as *k* = 147 bp. The size of the dimeric Pho4p is set as *k*_t_ = 17 bp. We vary the protein binding rate, *r*_onp_, relative to the nucleosome adsorption rate such that it has the following range: *r*_onp_ = [0.001, 1000] × *k*_0_*s*^−1^, where 
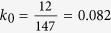
. The activator protein once bound can unbind from the DNA with intrinsic rates given by 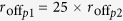. The constant of proportionality was calculated by comparing the probabilities of binding at UAS1 ≈ 3 × 10^−4^ and at UAS2 ≈ 7.5 × 10^−3^ as estimated by Lam *et al.*^38^. The values of the dissociation rate constants at binding sites UAS2 and UAS1 are given by 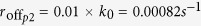 and 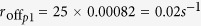, respectively. These values were adjusted according to the findings reported by Luo *et al.*^46^. In the LRA we fix *b* = 150 bp and *h* is kept as a variable. As previously described^47^, we assume the steady state maximal level of *PHO5* mRNA as 〈*m*〉 ≈ 12 per cell per transcriptionally active promoter state. Considering the half-life of an mRNA as *t*_half-life_ ≈ 5 min^48^, we can estimate the rate of transcription from active promoter states as, 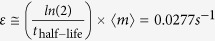, and accordingly the mRNA death rate becomes, 
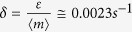
.

## Results

### Nucleosome occupancy in the *PHO5* promoter: Role of basal ATPase activity and DNA sequence

**Figure 3:**
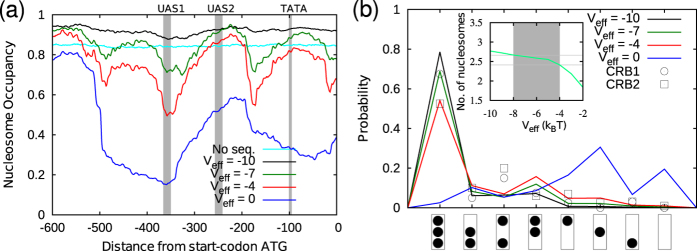
Nucleosome occupancy and promoter states in the absence of Pho4p. (**a**) Nucleosome occupancies for various amounts of basal ATPase activity designated as *V*_eff_. The cyan curve is for *V*_eff_ = −7 *k*_B_*T* with no sequence effects. Other curves are with sequence effects for *V*_eff_ = −10, −7, −4 and 0 *k*_B_*T* values (top to bottom). The Pho4p binding sites, UAS1 and UAS2, and the TATA box are indicated by grey strips. (**b**) Promoter-state distribution: In the X-axis, the eight implicit promoter states are depicted schematically as boxes with nucleosomes as dots—the top dot is nucleosome N − 1 and the bottom dot is nucleosome N − 3. The Y-axis gives the probability of finding these states. CRB1 (circles) and CRB2 (squares) are experimentally measured probabilities of these promoter states when the *PHO5* gene is repressed or inactive, as reported by Brown *et al.*^44^ (for details, see text). The experimental findings are compared with our simulation results obtained under the same conditions as in (**a**). The inset indicates the average number of nucleosomes for various *V*_eff_ values. The region −8 < *V*_eff_ < −4 is shaded (grey) to indicate nucleosome density of ≈75% to 90%.

First, we have simulated the nucleosome positioning in the *PHO5* promoter region taking into account only two factors—the effect of DNA sequence and the basal ATPase activity (Fig. 3a). In the absence of any sequence effects (homogeneous sequence, with only ATPase activity) we find that the positioning is absent, that is, uniform occupancy along the DNA (cyan, marked as “No seq.”). From the earlier studies^34^,^37^, it is expected that the basal ATP-dependent activity alone will not lead to positioning of nucleosomes. When sequence effect is accounted, nucleosomes get positioned according to the sequence (see *V*_eff_ = −7*k*_B_*T* curve and other curves). In the presence of sequence-dependent kinetics, the basal ATPase activity results in two effects: it controls the density of nucleosomes bound in the promoter region and it modulates the sequence-dependent positioning. For instance, the NFR at the UAS1 site (grey stripes in Fig. 3a) becomes more and more pronounced as ATPase activity (*V*_eff_) increases^26^. When ATPase activity is very low (viz. *V*_eff_ < −8*k*_B_*T*) the nucleosome density is very high, and the promoter is covered by nucleosomes.

To have a quantitative understanding of the number of nucleosomes bound in the promoter region and to examine how ATPase activity controls the nucleosome organization in promoters, we compared our simulation results to the known experimental observations obtained for the “inactive” (OFF/repressed) gene. It has been reported, experimentally, that the average number of nucleosomes in the promoter region, in repressed states, is between ≈2.4 and 2.65^44^. We find that these average numbers can only be obtained when the ATPase activity is ≈−4*k*_B_*T* > *V*_eff_ > −8*k*_B_*T* (Fig. 3b inset, Supplementary Fig. S1). This amount of ATPase activity gives us a sensible global nucleosome density of ≈75% to 90%, which is comparable to the density known in the literature^1^ (Supplementary Fig. S2). We further compared our results with the data obtained by Brown *et al.*^44^, where they have measured the individual probability of eight different spatial organization of nucleosomes in the *PHO5* promoter region (Fig. 2a, right; the eight “implicit” promoter states shown in the figure are the eight states observed by Brown *et al.*^44^). We computed the probability of finding these eight states for various amounts of ATPase activity (Fig. 3b). When the ATPase activity is either too high (*V*_eff_ > −4*k*_B_*T*) or too low (*V*_eff_ < −8*k*_B_*T*) (beyond the range discussed above), the spatial organization of the promoter nucleosomes (curve from simulation) is very different from the experimental data (squares and circles)—e.g., *V*_eff_ = 0*k*_B_*T* (blue curve) and *V*_eff_ = −10*k*_B_*T* (black curve). The CRB1 data points (circles) represent promoter configurations for constitutively inactive *PHO5* gene in *pho4*Δ*pho80*Δ*tata* mutant cells. The data points marked as CRB2 (squares) represent promoter for *pho*2Δ mutant cells grown under the repressing condition of a high phosphate growth medium. Except for the third and fourth states, all the probabilities of states obtained for *V*_eff_ = −7*k*_B_*T* and *V*_eff_ = −4*k*_B_*T* are comparable with CRB1 and CRB2 data points respectively (Fig. 3b). Note that these experimental data obtained for the mutants are also very similar to the results obtained for the wild type cells treated with high phosphate concentration^44^. A common feature observed among these repressed cells is that the promoter states that are filled with nucleosomes have high probability and promoter states that are depleted of nucleosomes have low probability. When the ATPase activity is very high, namely, *V*_eff_ > −4*k*_B_*T*, we found that nucleosomes are severely depleted. For instance, the density decreases as low as ~45% for the removal rate corresponding to *V*_eff_ = 0 (Supplementary Fig. S2). Evidently this would make promoters highly accessible to TFs and highly conducive to transcription. In other words, repressed states have relatively low ATP-dependent nucleosome removal activity and deviation from this may lead to unregulated/constitutive expression.

### Binding of transcription factor Pho4p alone cannot activate the gene

**Figure 4:**
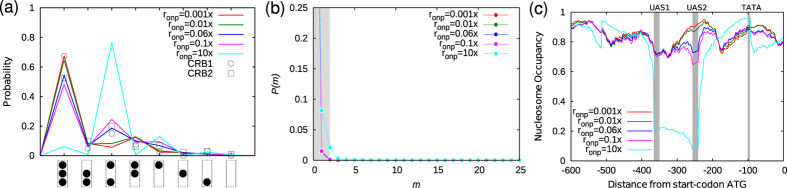
Repressed promoter in the presence of Pho4p. (**a**) Promoter-state distributions of the eight states as described in Fig. 3b. Pho4p binding is introduced with small binding rates, *r*_onp_ ≈ 0.001–0.1 × *k*_0_*s*^−1^ as well as with high binding rate, *r*_onp_ = 10 × *k*_0_*s*^−1^. In the legends, *r*_onp_ are expressed in units of *k*_0_ per second. (**b**,**c**) are the corresponding mRNA distributions and nucleosome occupancies, respectively. In (**b**), grey-shade is provided to indicate the fraction of the cells that are in the OFF state. All simulations were performed for *V*_eff_ = −7*k*_B_*T* and LRA, *h* = 0*k*_B_*T*.

As a next step, we introduced Pho4p binding kinetics (see Models and Methods; only Pho4p binding, no LRA i.e., *h* = 0*k*_B_*T*) and computed the probabilities of different promoter states (Fig. 4a). Interestingly, a small amount of Pho4p binding (*r*_onp_ ~ 0.1 × *k*_0_*s*^−1^) makes our curves comparable to the experimental data (CRB1 and CRB2) by redistributing the frequencies of the first, third, and fourth promoter states. One can understand this by noting the following: as the TFs start competing with nucleosomes, the −2 nucleosome region—the region corresponding to the UAS1 and UAS2—gets depleted of nucleosomes and the probability of the third promoter state increases. This is a direct consequence of competition between TFs and nucleosomes. Our study suggests how stochastic switching from promoter state 1 to state 3 is modulated by competitive binding of Pho4p. As seen here, in this range of low Pho4p binding rates, the promoter is shown to be inactive. The above results also suggest another interesting point: the non–uniform nature of probabilities seen here (in the repressed state) is due to an interplay between basal remodeling activity, DNA sequence-effcts and effects due to “barrier”-like non–histone proteins binding. In the absence of sequence effects and TF binding, one would obtain same probability for different 2-nucleosome promoter states (i.e., states 2, 3 and 4) and single-nucleosome promoter states (i.e., states 5, 6 and 7). To quantify the inactive promoter we investigated mRNA production and its statistical distribution. To do this, we extended our simulations and introduced mRNA production into the model—whenever the promoter is in one of the active or ON states, mRNA will be produced with a given rate (see Models and Methods for details). Reflecting the inactive nature of the promoter, the mRNA distribution is found to be skewed towards a major peak at zero (Fig. 4b).

To understand the inactive state and the influence of TF binding on nucleosome organisation, we computed nucleosome occupancy (Fig. 4c). The increase in probability of the third promoter state suggests that the TF binding reduces the nucleosome occupancy at the −2 region. It is interesting to note that a small amount of TF binding does not affect the occupancy at the TATA region considerably. However, a strong binding of TFs at UAS1 and UAS2 will create a visible NFR in the middle of the promoter; this will induce strong statistical positioning of nucleosomes that will ensure high occupancy at the TATA region (cyan curve, Fig. 4c). Contrary to a naive expectation that the TF bindings will disrupt the promoter nucleosome organisation, a strong TF binding is predicted to lead to high nucleosome coverage of the crucial TATA box (and hence, the inactive promoter). This high coverage is a result of multi–particle correlations between nucleosomes and TFs arising due to steric interactions and entropic effects (dynamics leading to all possible microstates). Emergence of these correlation effects are unique features of our model—they will not emerge out of earlier models for *PHO5* that do not explicitly account for TF and nucleosome dynamics.

Although we obtained a good understanding of the inactive state, these results so far suggest that such a simple model—a model with only basal ATPase activity, DNA sequence, and Pho4p binding—cannot reproduce the experimentally observed promoter states when the gene is transcriptionally active (compare active (CRBact) and inactive (CRB1 and CRB2) promoter states in Supplementary Fig. S3a).

### Local remodeling activity coupled with Pho4p binding dictates dynamics of genetic switch

**Figure 5:**
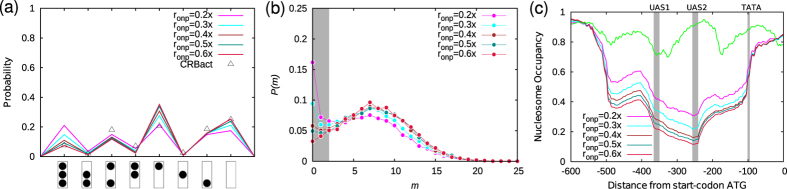
Active promoter with local remodeling activity. (**a**) Promoter-state distribution for selected Pho4p binding rate, *r*_onp_ ≈ 0.3–0.6 × *k*_0_*s*^−1^, that are the best fit with the experimental data, CRBact (triangles). CRBact data points were obtained from transcriptionally active *PHO4 pho80*Δ cells where Pho80p has lost its phosphorylation activity^44^. (**b**,**c**) are the corresponding mRNA distributions and nucleosome occupancies. In (**b**), grey-shade denotes the percentage of OFF cells, otherwise ON cells. Green curve in (**c**) is the occupancy in the repressed state. The data presented here are for *V*_eff_ = −7*k*_B_*T* and LRA, *h* = 21*k*_B_*T*.

We now address the following question: what is the nature of the underlying nucleosome dynamics that would generate the experimentally observed nucleosome pattern in the so-called “active” (ON) state of the promoter? So far, in our simulations, we have accounted for basal ATPase activity and Pho4p binding. However, from the high coverage of TATA discussed above and from Supplementary Fig. S3a, it is clear that the combination of basal ATPase activity and Pho4p binding alone cannot reproduce the experimentally observed “active” promoter-nucleosome patterns—no matter how much we increase the Pho4p binding rate, one will not be able to achieve the highly nucleosome depleted states (states from 4 to 8). Given that we have accounted for the sequence-dependent effects, BRA, and Pho4p binding already, the missing factor that may be crucial is the extra LRA present in the promoter region. In the literature it is known that the transcription activators, apart from physically occupying DNA, also recruit remodeling machines locally and induce removal of nucleosomes around its region of occupation^49^,^50^. We model this as an enhanced nucleosome removal activity concentrated only around the Pho4p binding sites by introducing a single parameter “*h*”. We define *h* as the maximal remodeling energy from bound Pho4p (within *b* = 150 bp, see Fig. 1) and it specifies the strength of the LRA, *V*^*r*^(*h*), as discussed in the model section (Fig. 1). Our simulation results, with LRA, are shown in Fig. 5 for different values of the protein binding rate, *r*_onp_, with *h* = 21*k*_B_*T* and *V*_eff_ = −7*k*_B_*T*. The probability distribution of the promoter-state obtained from the simulation is close to the observed experimental data (CRBact, triangles) obtained for constitutively active *PHO4 pho80*Δ cells, as reported in ref. 44 (Fig. 5a). Note that the rate of Pho4p binding that produces active promoter states falls in the range, *r*_onp_ ~ 0.3–0.6 × *k*_0_*s*^−1^ (Fig. 5a and Supplementary Fig. S3b). Interestingly, we also find that for LRA, *h* = 21 *k*_B_*T*, the mean dwell time (time for which a nucleosome is bound) of promoter nucleosomes ~0.1 *s*, which corresponds to nucleosome removal rate *r*_off_ ~ 10 *s*^−1^ (Supplementary Fig. S4). In other words, in order to obtain active promoter states, the activator-induced rate of nucleosome removal at the promoter must be close to the nucleosome adsorption rate, i.e., *r*_off_ ~ *r*_on_.

To further quantify the active state of the promoter, we investigated mRNA production and its statistical distribution. We found that mRNA expression, for parameters corresponding to the active state, shows a bimodal distribution (Fig. 5b)—one major peak near mRNA number *m* ≈ 10 representing cells in the ON state, and another peak near mRNA number *m* = 0 indicating a population of cells in the OFF state (Fig. 5b in grey-shade). This is a testable prediction of our model. The corresponding nucleosome occupancies in the promoter region are shown in Fig. 5c. Note that in the active state, the promoter is deprived of nucleosomes to a large extent (compare Fig. 5c with Fig. 3a; also see Fig. 4c). The nucleosome occupancy in the promoter region for the activator binding rate of ~0.3–0.6 × *k*_0_*s*^−1^ is about 40%. These data (Fig. 5b,c) indicate that transcriptionally active *PHO5* promoter show considerable heterogeneity in nucleosomal organization on the promoter^44^,^51^. The average nucleosome number (~1.15) is also comparable to the known experimental observation (~1.18)^44^,^47^. In Supplementary Figure S5, the time evolution of the promoter nucleosome number, the mRNA mean, and standard deviation are presented for LRA, *h* = 21 *k*_B_*T*.

### Local remodeling activity affects mRNA expression and transcriptional noise

**Figure 6:**
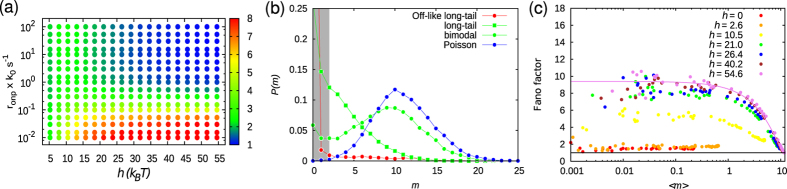
Effects of local remodeling activity on mRNA expression. (**a**) Phase plot of Fano factor, 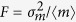, as a function of activator binding rate, *r*_onp_, and LRA, *h*. The color gradient on the right represents the value of *F*. (**b**) mRNA distributions: OFF-like long-tail (red), long-tail (green-square), bimodal (green-circle), and Poisson (blue). (**c**) Plot of Fano factor as a function of mRNA abundance, 〈*m*〉, for different values of *h* in the unit of *k*_B_*T*. Each point in the plot is obtained by keeping *h* constant and varying the protein binding rate, *r*_onp_. The data points were fitted using analytic functional form of Fano factor derived for a two-state random telegraph model: 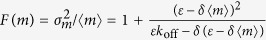, where *k*_off_ is the transition rate constant from active to inactive state. Here *k*_off_ = 0.001 *s*^−1^. All the simulations were conducted with *V*_eff_ = −7 *k*_B_*T*.

In this section, we systematically investigate the effect of Pho4p binding and LRA on mRNA expression and distribution. The nature of distribution can be characterised by defining the Fano factor, a commonly used gene expression noise parameter, as 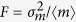, where 〈*m*〉 and 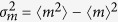 are the mean and variance in the mRNA expression, respectively. For Poisson distribution, *F* = 1. We construct a phase diagram by varying protein binding rate, *r*_onp_, and LRA parameter, *h*, and measure *F* (Fig. 6a). For some of the representative points in the phase diagram, we present the corresponding mRNA number distribution in Fig. 6b. From the phase diagram, it is clear that as a function of these two parameters, different kinds of expression patterns are possible (Fig. 6a).

For large LRA and high protein binding rate (*h* and *r*_onp_ are large), we find that the Fano factor *F* → 1 (blue region). This implies that the underlying process of gene expression has a Poisson distribution; this is indeed seen in Fig. 6b (filled circles). Since nucleosomes are highly depleted (high removal activity, *h* > 26*k*_B_*T*) and the protein binding is strong (*r*_onp_ > *k*_0_*s*^−1^), the probability of the promoter being in the ON state is also very high (*P*_ON_ > 90%). The corresponding gene expression is constitutive and we get high mRNA production. For other values of *h* and *r*_onp_, different types of non-Poissonian distributions can be obtained. The region given by the parametric values *h* ≥ 16 *k*_B_*T* and *r*_onp_ ~ 0.2–0.5 × *k*_0_*s*^−1^ (green region) displays bimodal distribution (Fig. 6b). In the bimodal case, there are considerable fractions of cells that are “ON” and “OFF” in the same population. The probability of the ON state in this case is *P*_ON_ ~ 50–70%. However, when *h* < 16 *k*_B_*T* (green region), the expression distribution is a monotonically decreasing function with a long-tail. In this case, the probability of having non-zero mRNA expression is high and decreases slowly for higher expressions. For protein binding rate *r*_onp_ < 0.1 × *k*_0_*s*^−1^ (red region), we obtained an OFF-like longtail distribution. In this phase-space, the promoter is OFF-like and has an expression with a very high probability of having no mRNA (OFF) and low or null probability of having non-zero mRNA (ON) (Fig. 6b; compare long-tial vs OFF-like long-tail). The probability of finding the cell in the ON state for the long-tail and OFF-like long-tail distributions are *P*_ON_ ~ 30% and *P*_ON_ < 10% respectively, suggesting that the genes are mostly OFF and the expression happens only rarely in short bursts. In Supplementary Fig. S6 we have also presented other intermediate distributions that arise as the parameters are varied.

To further check how LRA affects the dependence of noise strength, *F*, on the mean mRNA, 〈*m*〉, we calculated *F* as a function of 〈*m*〉 by using the protein binding rate, *r*_onp_, as the modulator to get the mean expression, 〈*m*〉 (Fig. 6c). The result clearly suggests that as LRA or *h* increases, the maximally attainable mean expression level, 〈*m*〉, also increases. Interestingly, for high LRA, *h* ≥ 26 *k*_B_*T*, the data points converge to the analytical Fano factor, *F*(〈*m*〉), derived for a two-state model of gene transcription when the promoter activation rate, say *k*_on_, is modulated (Fig. 6c). Incidentally, the value of the parameter used to fit our data is very small, *k*_off_ ~ 0.001 *s*^−1^, suggesting that our model can be represented by a two-state model by presuming promoter deactivation rate, *k*_off_, as a rate-limiting step. For *h* < 26 *k*_B_*T*, the Fano factor is low and 〈*m*〉 is also very low. For instance, as *h* → 0, 〈*m*〉 < 1 which is equivalent to no expression at all. For small 〈*m*〉 and non-zero values of *h*, the Fano factor is very large and fluctuates; however, as 〈*m*〉 increases, *F* → 1.

## Discussion

### Importance of the model

Despite the fact that there are studies trying to understand gene regulation in yeast promoters^47^,^52^, our work is novel for a variety of reasons: (1) We have accounted for the complete dynamics of nucleosomes and TFs explicitly, considering sequence-dependent and ATP-dependent kinetic moves. The introduction of sequence effects, for example, gives rise to less coverage of UAS1 compared to UAS2 (Fig. 3a). (2) We have considered nucleosomes/TFs as *extended* particles with steric hindrance. This gives rise to correlations in nucleosome/TF positions as seen in Fig. 4c. Such correlations will also give rise to cooperative effects in TF binding^53^,^54^. (3) We have considered the exposure of the TATA region (gene on/off), which is a kinetic process with multiple timescales based on local variation of sequence affinities and interaction with TFs. For example, disassembly/displacement of −1 nucleosome can happen in many different ways such that the TATA is exposed. Our simulations inherently account for all these different routes and this will affect the gene expression. (4) We can *simultaneously* predict nucleosome occupancy, nucleosome dynamics, accessibility of TF-binding and TATA sites, and the resulting gene expression. (5) Finally, our work fills a major gap in the literature to provide a combined understanding of nucleosome dynamics in the promoter region and in the coding region. The models that explain nucleosome positioning in the coding region cannot explain the nucleosome organization in the promoters^16^,^37^. We start from a basic model (see ref. 37) that can explain nucleosome organisation in the coding region, and go on to explain nucleosome organization in the promoter region. To do this, one crucial step we undertook is to separate ATPase activity into “basal” and “local”. Combining this current work with our earlier work^37^, we find that while BRA can explain nucleosome positioning at the coding region, TF-dependent LRA is absolutely necessary for the promoter region.

### Importance of nucleosome removal in the promoter region

The literature on nucleosome kinetics is dominated by nucleosome sliding^44^,^47^,^55^,^56^. However, our results suggest that nucleosome removal is the most important remodeling in the promoters. Without nucleosome disassembly, it is impossible to get promoter configurations that are completely free of nucleosomes.^44^. This fact is supported by our simulation results when only the sliding event is considered in the model (Supplementary Fig. S7). For high occupancy >75%, the probability that the promoter has no nucleosome is less than 0.25 (Supplementary Fig. S7, cyan and red curves), unlike in the experiments. Moreover, sliding of nucleosomes in the promoter region is likely to be hindered by the steric interaction of a number of non–histone proteins such as Pho4p. Pho4p will be bound in between the −1 and −3 nucleosomes restricting the sliding moves for all the three promoter nucleosomes (−1, −2, and −3). This suggests that nucleosome disassembly is the most likely way to obtain NFR in the inducible promoters^31^. In this paper, since we have nucleosome disassembly and sliding as separate kinetic events, we can obtain the experimentally observed nucleosome distribution even after accounting for the steric repulsion of the TFs.

### Competition between TFs and nucleosomes and local remodeling

From the current study we learn two things: (i) transcription factor binding alone may not be sufficient to switch ON the gene, and (ii) location-dependent remodeling is absolutely necessary. Pho4p binding can only occlude the −2 nucleosome. Therefore, one has to go beyond a passive model of activator binding, and a good candidate for this would be the recruitment model^49^, where the remodeling machineries are recruited to the promoters by TFs like Pho4p. These machines will evict nearby nucleosomes facilitating further recruitment of transcriptional machineries. The remodeling is local in the sense that it affects only the flanking nucleosomes^42^ lying within a region ≈150 bp from the Pho4p. Thus, well-positioned nucleosomes on promoters are evicted as a function of activator (Pho4p) concentration.

The precise role of steric exclusion by TFs in nucleosome remodeling is still needed to be fully understood. Brown *et al.* have suggested that there is no steric exclusion of nucleosomes by TFs, which means that nucleosome formation is independent of TF binding^44^. However, *in vitro* reconstitution experiments of *PHO5* promoter chromatin suggest that there is competition between Pho4p and nucleosome^57^. Our findings support the hypothesis of steric exclusion and provide further insights into the competition between TFs and nucleosomes. However, further studies are needed to have a better understanding of the contribution of steric hindrance by TFs in nucleosome positioning.

### Transcriptional noise and mRNA distribution

We present one of the first computational studies that explicitly incorporates nucleosome and TF dynamics accounting for DNA sequence-dependent and ATPase effects. Through this study we show how nucleosome dynamics coupled with TF binding kinetics can give us gene expression profiles that capture most of the essential features of a typical eukaryotic gene expression. By tuning the parameters like LRA and TF binding rate, we can get the Poisson, bimodal, long-tail, OFF-like long-tail, and other intermediate distributions. For sensible parameters (where nucleosome distribution is comparable with experimental data), we get a bimodal distribution for mRNA expression. Bimodal gene expressions are typically associated with inducible promoters^24^,^58^. The Fano factor for bimodal distribution is greater than unity implying that the gene expression is bursty and has a larger variance compared to the Poissonian or “constitutive” gene expression. The dominant cause of expression variability has been shown to be related to the nucleosomal promoter variations^51^,^59^. Since the basal expression associated with Pho4p-less promoter states is generally very low as compared to the activated expression^44^, the basal contribution to mRNA distribution is neglegible (see Supplementary Fig. S8). Taken together, our results give insights into how different factors, such as nucleosome-remodeling and TF binding, might affect the transcriptional noise and mRNA level.

Even though, in this paper, we have compared our results with the data of Brown *et al.*^44^, there exist other experimental studies that investigate nucleosome positioning in the *PHO5* promoter. In a recent study by Small *et al.*^51^, using a technique of methylating unprotected GpCs on the genome, they inferred various *PHO5* promoter states. They show that there is a redistribution of the state probabilities upon altering cellular conditions from a nutrient rich (repressed) to phosphate starvation (induced)—the former has states with many nucleosomes while the latter is predominantly depleted of nucleosomes. Broadly, even our simulations agree with this picture where the inactive state is dominated by bound nucleosomes and the active state is relatively nucleosome free. However, one of the major differences between the Small *et al.* data and Brown *et al.* data is that, in the Small *et al.* experiments, UAS1 is covered with nucleosomes more frequently, even in the active state. We find that we can obtain this feature by appropriately modifying the LRA (see Supplementary Fig. S9). The interesting aspect is that both these sets of experiments can only be explained if we incorporate transcription factor-mediated local remodeling and steric exclusion, which is the key conclusion of our paper. Also note that, even though we have chosen a set of sensible parameter values for the rates of nucleosome dynamics, average number of mRNA^60^, etc., it is possible that a different combination of parameter values may exist *in vivo*, depending on different cellular conditions. However, our results would not depend on the exact values of the parameters as the outcomes in this paper are guided by general physical principles such as the nucleosome removal, accessibility of DNA due to nucleosome depletion, and the LRA.

### Suggestion for new experiments to test our predictions

In order to test our predictions in this paper, we suggest a set of experiments that can be performed: (i) One can test our prediction for nucleosome occupancy shown in Fig. 3 by measuring *in vitro* nucleosome occupancy at low ATP concentrations and/or in the presence of mutated remodeling enzymes (*in vivo*). Under these conditions, one can also vary Pho4p expression (overexpress) and measure nucleosome occupancy in the *PHO5* promoter (Fig. 4). Another clear test of our model and results will be to test the importance of sequence-dependence; we predict that sequence-dependence will lead to variation in probabilities within 1- or 2-nucleosome promoter states(Fig. 4). This may be tested by repeating similar experiments (in a repressed-like condition) taking different sequences. (ii)The predictions in Fig. 6 regarding mRNA distributions can be tested with appropriately designed experiments. We predict how Fano factor and mRNA distributions would change as a function of LRA. Experiments may tune LRA in different ways: by varying the ATP concentration or by genetic deletion/mutation of genes that encode remodeling complexes, or by overexpression of these enzymes. We predict that mutations in remodeling enzymes or decreasing the ATP concentration will result in mRNA expression with long-tail distributions; on the other hand, overexpression of remodeling enzymes will lead to mRNA expression with Poisson distribution. (iii) The role of LRA and Pho4p on distribution of promoter states can be also be tested by carrying out experiments that involve isolation of chromatin rings^44^,^59^ from cells under various conditions of LRA and activator concentration as mentioned above.

In summary, we have studied a computational model for promoter dynamics of the *PHO5* gene in *Saccharomyces cerevisiae*. In the model, we include nucleosome and Pho4p dynamics taking into account the effects of DNA sequence and ATP-dependent remodeling activity. We first validate our model by making sure that our results are comparable with known experimental data of promoter states having different nucleosome configurations. Then we go on to predict a number of features such as the necessity for LRA with nucleosome removal. We show that only the binding–dissociation interaction of Pho4p with DNA does not give rise to active promoter states; we require Pho4p-mediated “local remodeling activity” on promoter nucleosomes by ATP-dependent remodeling complexes. We also show that the mRNA expression profile for high LRA is a Poisson distribution, and for low LRA the expression is a non-Poisson distribution.

## Additional Information

**How to cite this article**: Kharerin, H. *et al.* Role of transcription factor-mediated nucleosome disassembly in *PHO5* gene expression. *Sci. Rep.* **6**, 20319; doi: 10.1038/srep20319 (2016).

## Supplementary Material

Supplementary Information
