# Corrigendum: Role of transcription factor-mediated nucleosome disassembly in *PHO5* gene expression

**DOI:** 10.1038/srep22220

**Published:** 2016-03-04

**Authors:** Hungyo Kharerin, Paike J. Bhat, John F. Marko, Ranjith Padinhateeri

Scientific Reports 6: Article number: 20319; 10.1038/srep20319 published online: 02 04 2016; updated: 03 04 2016.

This Article contains errors in the Acknowledgments section.

“JFM acknowledges support by NSF Grants DMR-1206868 and MCB-1022117, and by NIH Grant DMR-1206868.”

should read:

“JFM acknowledges support by NSF Grants DMR-1206868 and MCB-1022117, and by NIH Grants R01-GM105847 and U54-CA193419 (CR-PS-OC).”

