# Supplementary material for: Role of transcription factor-mediated nucleosome disassembly in PHO5 gene expression
Source: Sci Rep. 2016 Feb 4;6:20319. doi: 10.1038/srep20319 (PMC4740855; doi:10.1038/srep20319)
Supplement: Supplementary Information [file srep20319-s1.pdf]

Supplementary Information

Role of transcription factor-mediated nucleosome disassembly in *PHO5* gene expression

Hungyo Kharerin, Paike J. Bhat, John F. Marko, and Ranjith Padinhateeri

Rules for identifying promoter states

Brown et al. have identified eight distinct promoter-nucleosome states from their recent experiments [1]. They identified three important nucleosome positions – N-1, N-2 and N-3. Similar to their approach, we also define three nucleosome locations namely N-1 (also known as -1 nucleosome), N-2 (also known as -2 nucleosome) and N-3 (also known as -3 nucleosome) as follows:

N-1 is the nucleosome that covers the TATA site at -98 bp,

N-2 is the nucleosome that covers the UAS2 site at -249 bp,

N-3 is the nucleosome which lies upstream of UAS1 that covers -460 bp.

These positions are measured with respect to start-codon ATG as the coordinate origin.

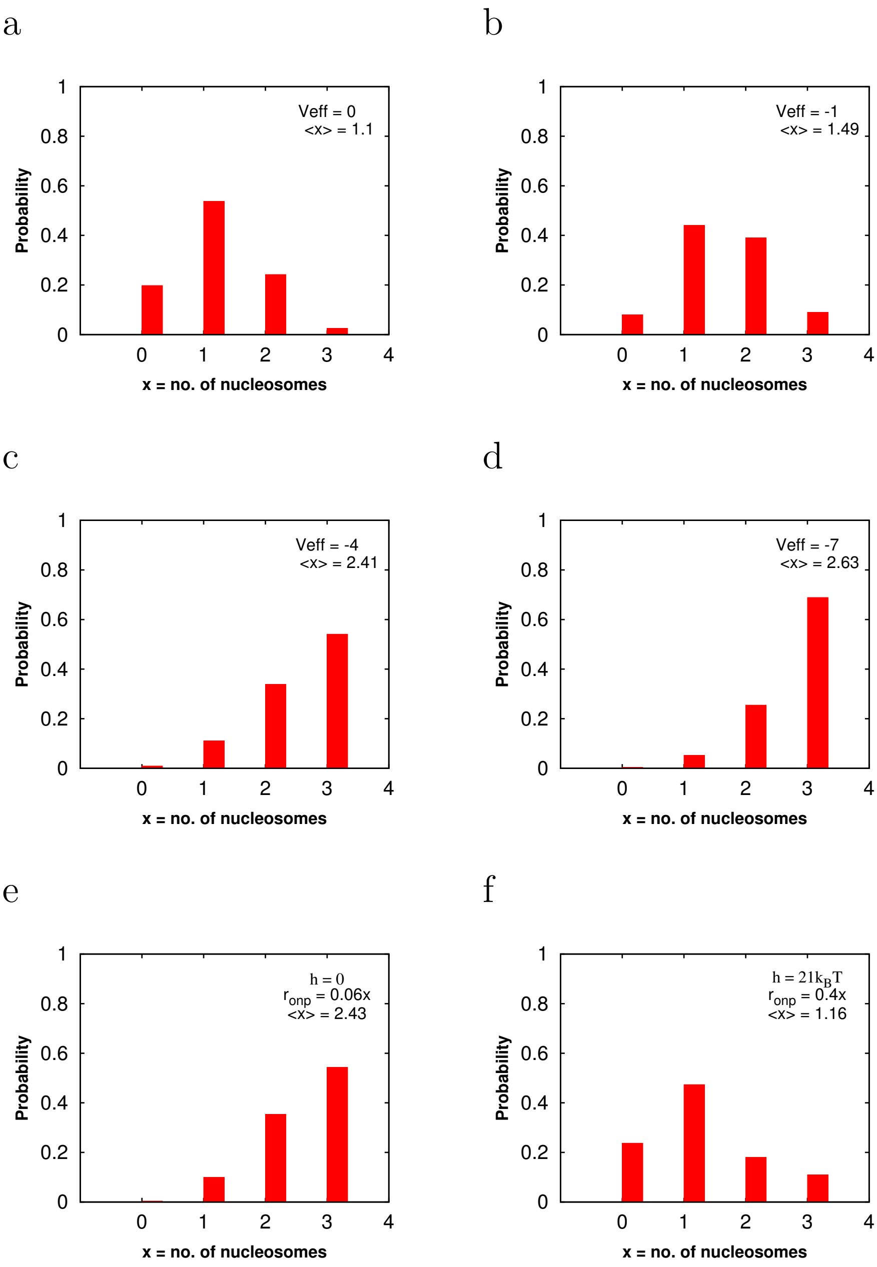

Figure S1: Histogram of nucleosome number at the promoter region (600 bp upstream of start-codon ATG) for various values of effective potential, V_{eff}. (a-d) Nucleosome number in the absence of activator protein, Pho4p, and local remodeling activity (LRA). (e,f) Nucleosome number in the presence of Pho4p and LRA. (e) shows repressed condition when LRA, $h = 0 k_{\text{B}}T$, and (f) shows induced condition with $h = 21 k_{\text{B}}T$. Here $V_{\text{eff}} = -7 k_{\text{B}}T$.

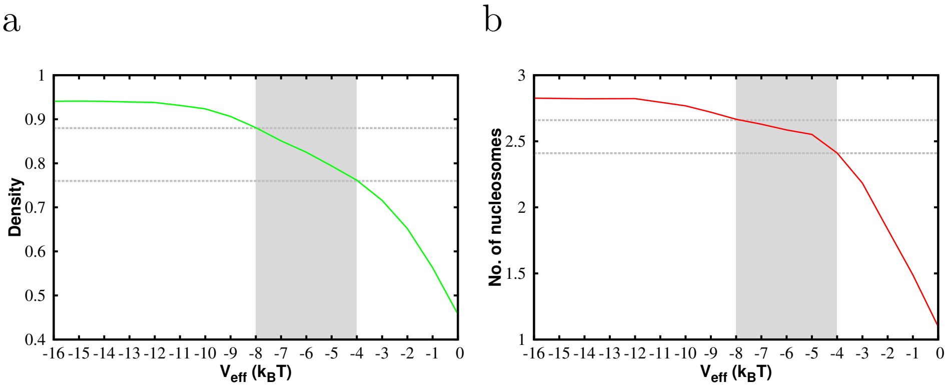

Figure S 2: Global nucleosome density (a) and promoter nucleosome number (b) in the absence of activator proteins. All the data points and calculations were made when equilibrium occupancy is established (after $\sim$ 1hr of simulations). The shaded region from $V_{\rm eff} = -8k_{\rm B}T$ to $-4k_{\rm B}T$, with corresponding densities from 88% to 76% (left), and the corresponding promoter nucleosome from ≈ 2.66 to 2.41 (right, red curve), presumably falls in the range of biologically relevant densities. For $V_{\rm eff} = -7k_{\rm B}T$, the global nucleosome density is $\approx 85\%$ and the corresponding number of nucleosomes in the promoter (600 bps) is ≈ 2.63 (Fig. S1d).

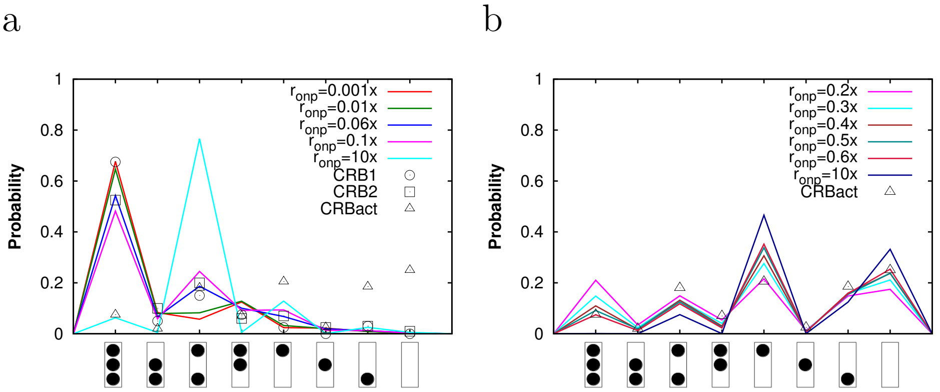

Figure S 3: Distribution of promoter states for various activator binding rates, $r_{\rm onp}$, when LRA is (a) $h = 0k_{\rm B}T$ and (b) $h = 21k_{\rm B}T$. The data points given by CRB1, CRB2 and CRBact are experimentally obtained results [1] (see the main text).

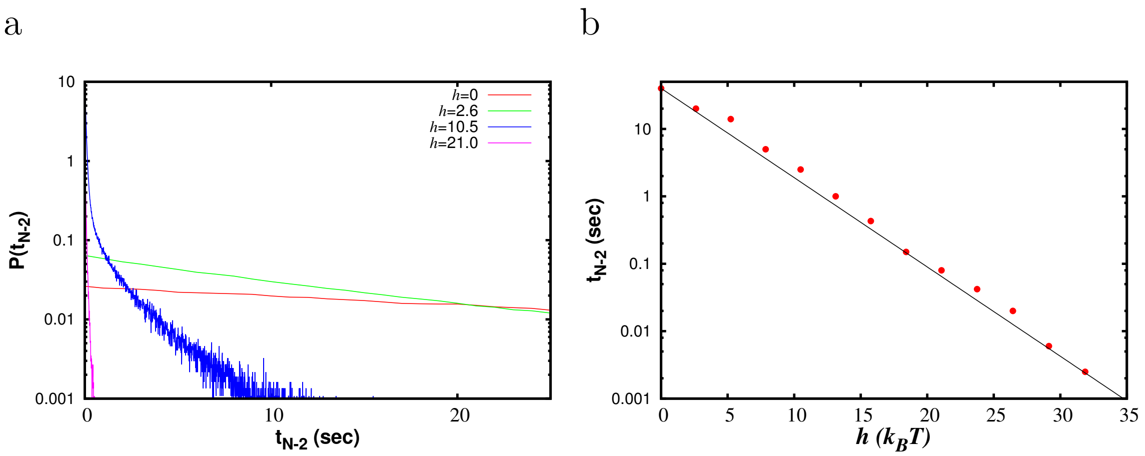

Figure S4: Timescales for nucleosome removal in the presence of LRA. (a) Distribution of first dwell times for nucleosome N-2 in the presence of LRA. (b) Reciprocals of the slopes calculated at long timescales obtained from distributions given in (a) are approximated as the mean first dwell times and plotted as a function of h (red) with exponential fit (black).

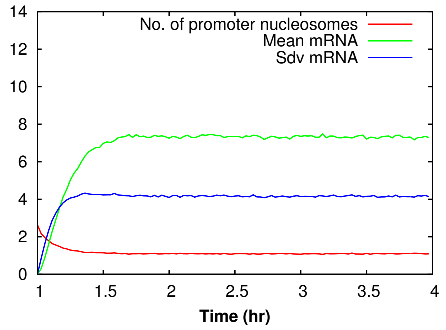

Figure S5: Time evolution of nucleosome number (red) in the promoter, mean (green) and standard deviation (blue) of mRNA as function of time for activator binding rate, $r_{\rm onp} = 0.5 \times k_0 s^{-1}$, with LRA, $h = 21 k_{\rm B}T$.

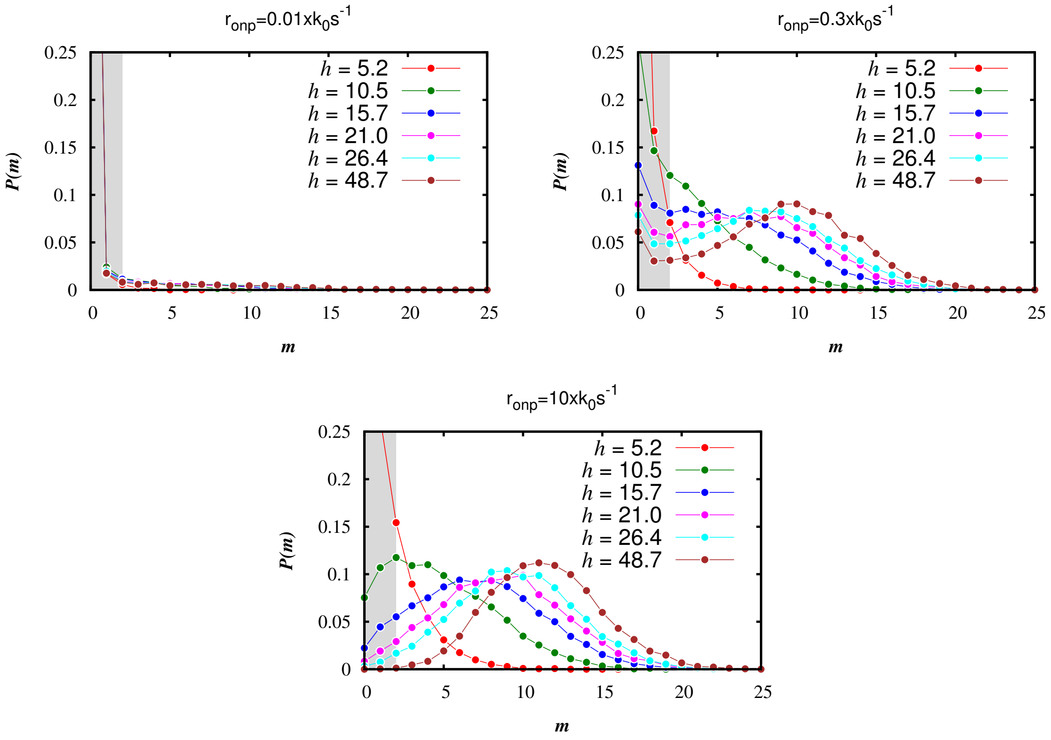

Figure S6: mRNA distributions for various values of activator binding rates (r_{onp}) and LRA (h) (see Figure 5a in the main text).

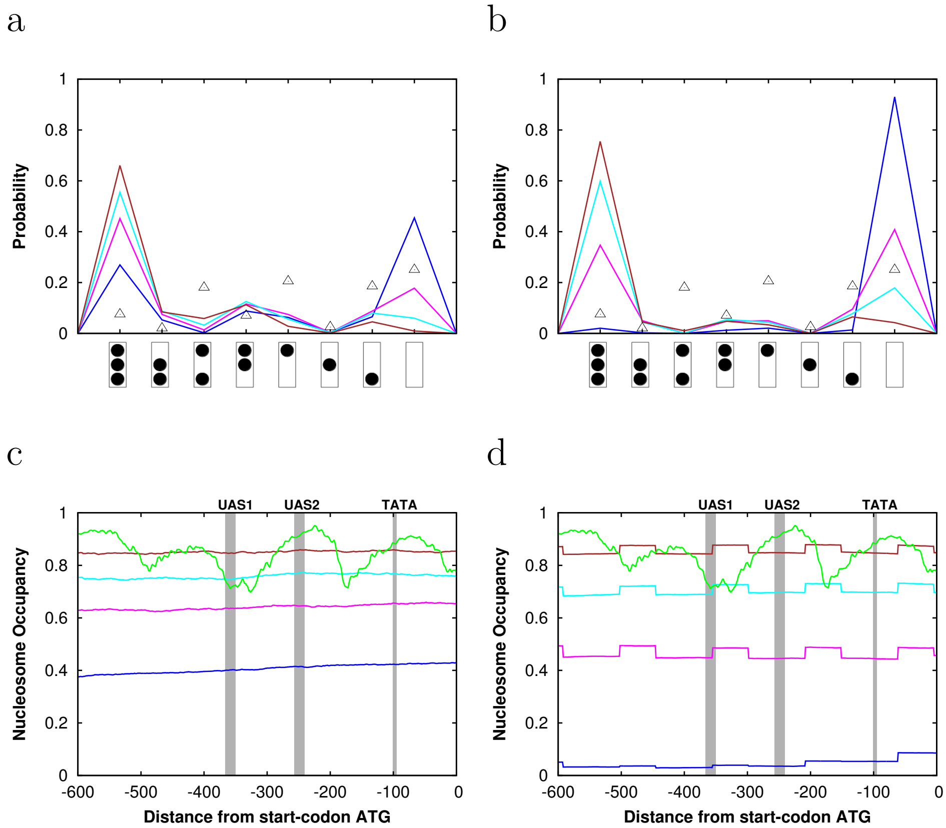

Figure S 7: Distribution of promoter states (a,b) for corresponding nucleosome occupancies (c,d). For example in (c), the occupancies are 40%(blue), 65%(pink), 75%(cyan) and 85%(red). These data were generated by taking into account only the nucleosome sliding event and no activator binding in the model, except for the green curve in (c,d), which was calculated for $V_{\text{eff}} = -7k_{\text{B}}T$ representing occupancy of a transcriptionally repressed promoter. (a,c) is simulated using sliding rate, $\alpha = 0.0024s^{-1}$, and (b,d) is simulated using $\alpha = 0.024s^{-1}$. The data points represented by triangles in (a) and (b) are experimentally obtained results for constitutively active *PHO5* promoter [1] (see the main text).

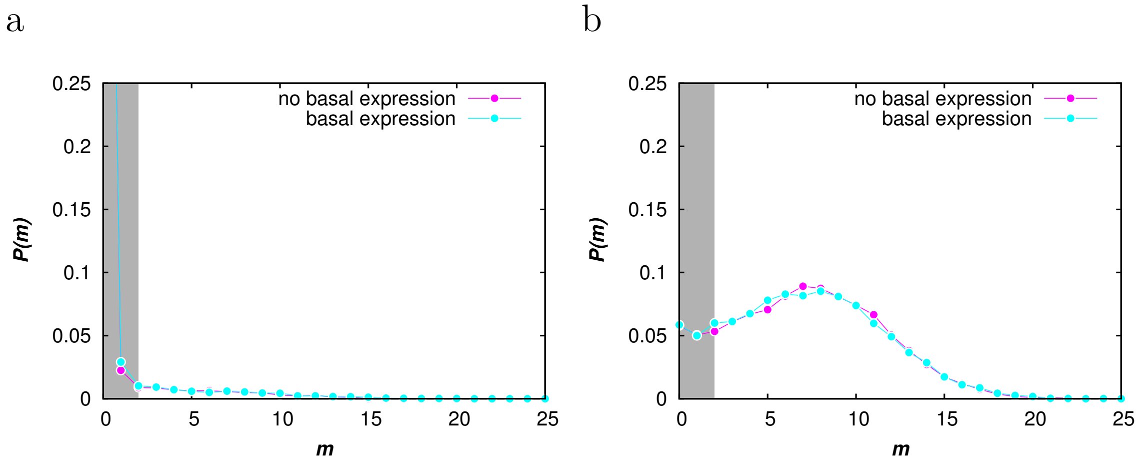

Figure S 8: mRNA distribution with (pink) and without (cyan) basal expression for activator binding rates (a) $r_{\rm onp} = 0.01 \times k_0 s^{-1}$ and (b) $r_{\rm onp} = 0.4 \times k_0 s^{-1}$. The rate of basal transcription and the rate of activated transcription are $\epsilon_{\rm b} = 0.00027 s^{-1}$ and $\epsilon = 0.0274 s^{-1}$, respectively, such that the mean mRNA per induced cell is 12 molecules. These are calculated for $V_{\rm eff} = -7 k_{\rm B}T$, and local removal activity, $h = 21 k_{\rm B}T$.

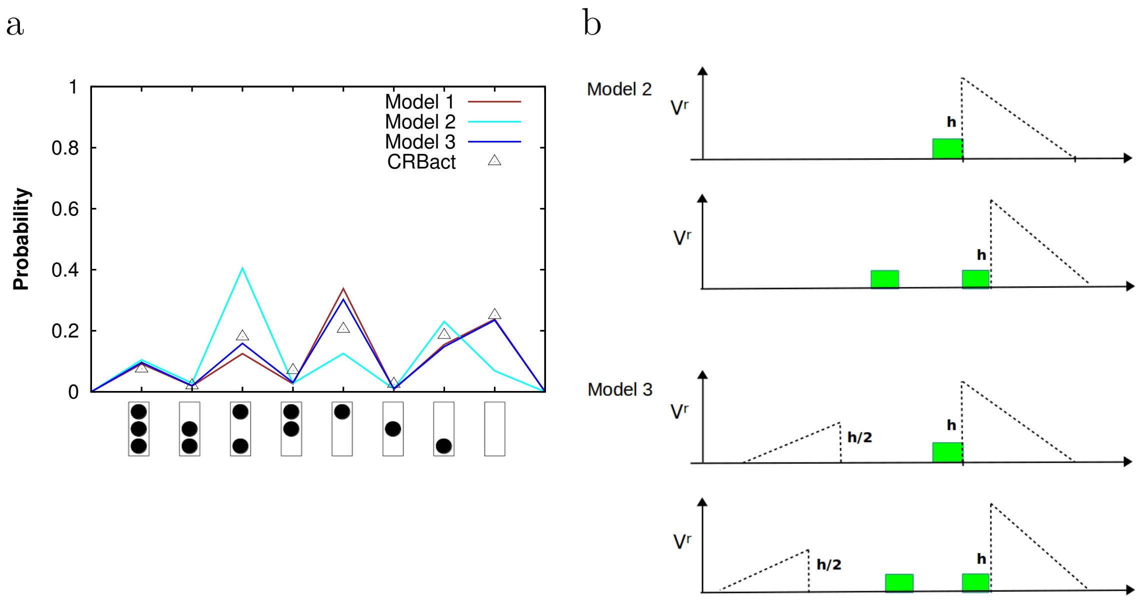

Figure S9: Comparison of various LRA models. (a) Distribution of promoter states under various models for LRA. Model 1 is same as the LRA model presented in Fig. 1c and d, in the main text. Model 2 and 3 are alternative LRA models shown in the adjacent figure (b). CRBact is the experimental data for transcriptionally active cells. (b) Model 2 is when remodeling activity is present only on the downstream of UAS's. Model 3 is similar to Model 1 (remodeling activity present on the upstream as well as on the downstream of UAS's) except that the upstream remodeling activity is less than the downstream remodeling activity.

[1] Brown CR, Mao C, Falkovskaia E, Jurica MS, Boeger H (2013) Linking stochastic fluctuations in chromatin structure and gene expression. *PLoS Bio.* 11.
